# Using Targeted mHealth Messages to Address Hypertension and Diabetes Self-Management in Cambodia: Protocol for a Clustered Randomized Controlled Trial

**DOI:** 10.2196/11614

**Published:** 2019-03-19

**Authors:** Annette L Fitzpatrick, Maurits van Pelt, Hen Heang, Lesley Steinman, Nicole Ide, Chhorvann Chhea, James P LoGerfo

**Affiliations:** 1 Department of Family Medicine University of Washington Seattle, WA United States; 2 Department of Epidemiology University of Washington Seattle, WA United States; 3 Department of Global Health University of Washington Seattle, WA United States; 4 MoPoTsyo Patient Information Center Phnom Penh Cambodia; 5 Department of Health Services University of Washington Seattle, WA United States; 6 National Institute of Public Health Phnom Penh Cambodia

**Keywords:** Cambodia, diabetes, hypertension, mHealth, mobile phone

## Abstract

**Background:**

Hypertension and diabetes represent the first and third highest contributors to global disability. While mobile health (mHealth) messaging programs have rapidly increased in low- and middle-income countries (LMIC), adaptations for specific patient health needs is a new approach to manage chronic conditions.

**Objective:**

The primary aim of this study is to develop and test an mHealth communication intervention using electronic data capture (by tablet) and voice messaging to improve hypertension and diabetes self-management in Cambodia. The secondary aim is to share results with the Cambodian Ministry of Health and development partners to inform health policy and develop strategies for hypertension and diabetes control.

**Methods:**

The study design is a cluster randomized controlled clinical trial randomizing each of 75 Community peer educators (PEs), trained and coordinated by MoPoTsyo Patient Information Center in Phnom Penh, into one of 3 groups of 25 (approximately 60 patients each) to receive either tablet+messages, tablet only, or no intervention (control). The total sample within each group includes 25 clusters and approximately 1500 patients located in 7 Operational Districts in rural regions or urban slums in Cambodia. The interventions (groups 1 and 2) were compared with usual PE monitoring without the tablet or mHealth messaging interventions. Focus groups and informant interviews were conducted to develop messages according to specific themes—medications adherence, laboratory testing, physician visits, obesity, smoking, and general lifestyle issues. Using the data received at monthly PE monitoring meetings, patients will receive specific messages based on their individual health challenges. Following the intervention completion, clinical and process outcomes will be compared with baseline metrics between groups.

**Results:**

PEs were randomized in July 2017, and the intervention was implemented in September 2017 through June 2018. Analyses are underway.

**Conclusions:**

This project is unique in its combination of electronic data transfer, which can be accessed immediately, with voice messages most relevant to individual patients’ needs. Positive results will indicate the value of using targeted messaging in patient-specific, self-management issues to improve hypertension and diabetes control.

**International Registered Report Identifier (IRRID):**

DERR1-10.2196/11614

## Introduction

The incidence and prevalence of cardiovascular disease and its risk factors, including hypertension and diabetes mellitus, is increasing rapidly in low- and middle-income countries (LMIC) [1-3]. After the turn of the century, epidemiological trends emerged that were characterized by a major shift in the size and relative magnitude of many risk factors for mortality and disability. High systolic blood pressure and high fasting plasma glucose increased steadily in impact on the global burden of disease [4]. Between 1990 and 2015, systolic blood pressure increased across the globe with associated increases in both mortality and disability [5]. In 2015, the top 3 greatest contributors to global disability-adjusted life-years among level 3 risks were high systolic blood pressure, smoking, and high fasting plasma glucose [6]; these increases are noteworthy as their sequelae, heart disease and stroke, are now the leading cause of death in these nations [7]. In Cambodia, it has been estimated that more than half (52.0%) of the total diabetic population is untreated, and despite treatment, only 24% of all diabetes is adequately controlled [8]. Poverty, inequality, lack of education, along with the nature of the nutritional transition, are root causes of the problem while limited resources mean that noncommunicable diseases (NCDs) must compete for political attention and financial investment. The global trend of increasing risk factors for cardiovascular diseases will continue unless effective methods are implemented to not only identify high-risk individuals but also implement effective and sustainable lifestyle and pharmacological interventions [9]. Tools and interventions to improve the self-management of cardiovascular disease risk factors, such as hypertension and diabetes, are greatly needed in community settings where health care resources may be limited.

Mobile health (mHealth) messaging programs have rapidly increased in LMIC and provide a means to support self-management programs especially needed for NCDs. A number of studies have focused on short message service (SMS) text messaging to improve outcomes for hypertension [10,11] and diabetes [11,12-14]. While these studies have generally shown messaging to be an acceptable format to participants, results have been mixed, with several showing small changes in some disease outcomes [10,12-14] or behavioral change [11,15]. However, no consistent patterns between intervention and control groups have been found. While mHealth messaging systems in the literature have varied in terms of features provided, the evaluation of targeting patients with voice messages to address individualized problems has been limited.

This study aims to leverage the activities of a Cambodian nongovernmental organization, MoPoTsyo Patient Information Center, to test if improvement of data transfer using electronic data capture combined with sending targeted voice messages to patients, would improve outcomes for hypertension and diabetes. As peer educators (PEs) were already established in the MoPoTsyo system to monitor hypertension and diabetes across the country, it provided us with the opportunity to both increase the time from patient measurement to data utilization (through e-tablets) and to use these data to individualize messages targeting specific issues revealed from the data (voice messages).

The primary aim of this study is to develop and test an mHealth communication intervention using electronic data capture and voice messaging to improve hypertension and diabetes self-management in Cambodia by implementing a combined electronic health (eHealth) and mHealth intervention. The secondary aim is to share results with the Cambodian Ministry of Health and development partners to inform health policy and develop strategies for hypertension and diabetes control.

## Methods

### Human Subjects Approval

This is a randomized controlled clinical trial to test an intervention comprising faster data capture plus targeted voice messages compared with PE monthly monitoring in a community-based sample in Cambodia for improvement of hypertension and diabetes outcomes. This study received Institutional Review Board approval from the University of Washington Division of Human Subjects and the National Ethics Committee for Health Research in Cambodia. All PEs, regardless of study allocation, and participants assigned to the telephone message intervention provided written informed consent. Informed consent from community participants who did not receive mHealth messages were waived by the Institutional Review Boards as routine monitoring provided study outcome data for these groups. This study was determined to not qualify as an “Applicable Clinical Trial” according to the National Institutes of Health definition [16] at the time it was initiated and, thus, was not registered in clinicaltrials.gov. As the risk of harm is minimal in this study, no criteria for discontinuation were developed. An Advisory Committee comprising Cambodian Ministry of Health officials, health care providers, and technology experts was convened to provide oversight and guidance to the study.

### Study Setting

In Cambodia, where greater life expectancy is causing rapid increases in NCDs [17], nongovernmental organizations have stepped in when government programs have not been able to address disease locally. MoPoTsyo Patient Information Center is a Cambodian nongovernmental organization for people with chronic disease in Cambodia [18]. It was established in 2004 to provide an institutional and practical response to the information and care needed by patients with hypertension and diabetes. Their model recruits and involves patients as volunteers and trains them to provide counseling on NCDs and monitor key health indicators over time. PEs see patients on a monthly basis to reinforce training and monitor key metrics, including blood pressure, glucose, weight, and adherence to medications. Patients keep paper logs of their health information that are updated at each PE visit. Currently, logs summaries are transferred to the MoPoTsyo Patient Information Center for manual entry and to update patient histories. Since 2005, MoPoTsyo has trained >200 PEs who have registered >31,000 patients in 7 rural provinces and poor urban slum areas of Phnom Penh. Patient and pharmacy records have been used to document the success of the program showing dramatic improvement in chronic disease management [19].

Seven Operational Districts (ODs) representing rural geographic regions or urban slums were selected for inclusion in the project; these included 4 ODs in Kampong Speu province, 2 ODs in Kampong Thom province, 1 OD in Kampong Cham province, and the municipality of Phnom Penh. This area includes 87 PEs who were included for simple randomization (by computer-generated random numbers) into the 3 arms of the study. All patients registered into the MoPoTsyo Patient Information Center system at the time of PE randomization were included as the community sample to receive mHealth messages with no exclusion criteria. Owing to the nature of this intervention, it was not possible to blind participants to study allocation. However, as patients of PEs were clustered geographically, there was a minimum chance of patients interacting across clusters to discuss the study.

### Approach

**Figure 1:**
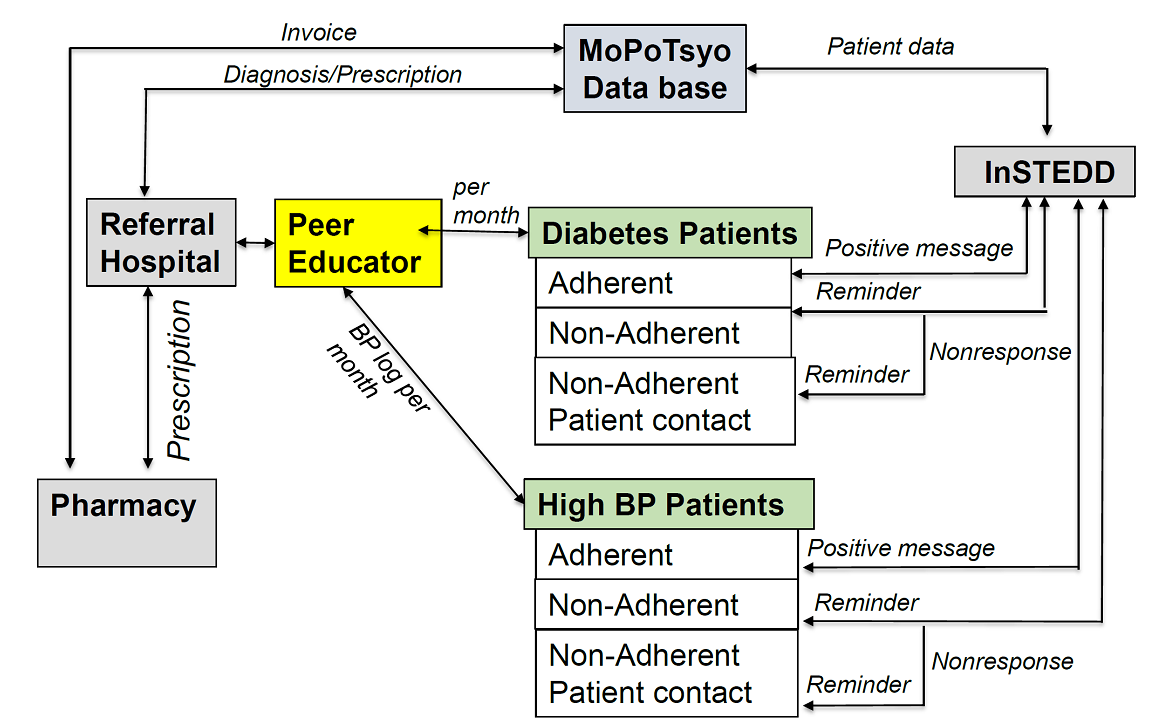
Study design of the project. BP: blood pressure; InSTEDD: Innovative Support to Emergencies, Diseases and Disasters.

In this study, we are collaborating with MoPoTsyo Patient Information Center to enhance their Peer Educator Network model through the application of mHealth-tailored phone messaging and improved eHealth communication throughout the system. A focus on hypertension, justified by the extremely low medication adherence of hypertensive patients compared with diabetic patients, shifts the intervention to an area of great need. Two interventions will be tested, one at the PE level and one at the patient level. The first includes providing electronic tablets to PEs for data collection and transfer to the MoPoTsyo database in Phnom Penh. The tablets are intended to speed up data collection and entry, reduce paper, increase accuracy, and eliminate lengthy distances that must be traveled to bring paperwork to MoPoTsyo. In addition, the tablets will allow PEs to scan a patient’s record “handbook” (log kept with the patient) to be verified by MoPoTsyo quality control staff should data appear suspicious. At the patient level, voice messages developed to address specific patient problems (eg, uncontrolled blood pressure or glucose, medications not picked up at the pharmacy, weight gain, etc) will be sent to patients based on the data received from the monthly visits to the PE. This study has randomly selected 75 PEs for assignment to 1 of 3 intervention groups—tablet+messaging, tablet only, or control (no tablet or voice messages). Figure 1 shows the study design. As we estimate that each of the 25 PEs in each group monitors about 60 patients, each arm of the study will include about 1500 participants for a total community sample of about 4500.

### Electronic Data Capture

Fifty PEs (25 in the tablet+messaging group and 25 in the tablet only group) received a 12-inch Chuwi Hi12 Tablet with touchscreen compatible for Windows 10-Android 5.1 to record patient information each month. Data are entered online or offline during the patient visit with backup recorded in the patient handbook (log). Data are transferred to the MoPoTsyo database stored on a Dell PowerEdge T630 Server, RAM 32GB (Gigabytes), HDD 4TB, (hard disk 4 Terabytes), speed 2.4 GHz, Cache 20MB. The operating system is Windows Server 2012R2 with database application Microsoft SQL server 2014. Initial training has been provided by MoPoTsyo staff to assure PEs are comfortable with using the tablets for their monthly monitoring visits. It has become clear, perhaps because of their older age and lack of experience, that many of the PEs are challenged by the technical aspects of this new tool. We have begun weekly Web-conferences (broadcast on the tablets) to help address problems and increase PE skills in tablet use. In addition, pharmacies have received tablets to input prescriptions and provide information to MoPoTsyo regarding invoices when medications are purchased. We do not anticipate significant dropout of PEs based on historical experience. PEs seldom leave MoPoTsyo unless illness or death intervenes. Retention efforts for community participants include the free health monitoring provided by PEs and the social interactions that result in this popular program. PEs in all groups are coached to be vigilant in responding to patient questions and needs as well as to contact the MoPoTsyo Center with any issues that arise.

### Voice Message Development

We used an exploratory qualitative study design to hear patient and PE perspectives on NCD management and mHealth. We used the 32-item checklist Consolidated Criteria for Reporting Qualitative Research [20] to guide our study design, data collection, analysis, and reporting of our research study. The Information-Motivation-Behavior theoretical framework was used to guide mHealth message development [21] based on providing accurate information, personal motivation, and social motivation to impact behavioral change in chronic disease self-management. We conducted focus groups (N=59) in Khmer with MoPoTsyo patients at 6 ODs within the study territory (Kampong Speu, Chamkar Leu, Baray-Santuk, Stoong, Kong Pisey, and Phnom Penh) to better understand the content of messages that would be most effective and provide guidance on logistics for sending them. Focus groups were held at community Health Centers and lasted for approximately 1.5 hours each. Focus group discussions were audiorecorded with participant permission. For the message content, the focus groups discussed current activities for managing NCDs (eg, medication use, doctor’s visits, lab tests and monitoring, PE groups, and lifestyle changes in diet, physical activity, smoking, and alcohol use), as well as facilitators and barriers to these activities. For the message format, questions included the frequency and duration of messages, preference for text or voicemail format, access to cell phones, the best time of day to send, and ability to respond to a text. At each district, 2 PEs (convenience sample) were also interviewed to evaluate features of the tablet for tracking their patients, to provide outreach and assure usability.

The taxonomy of behavioral change communication deemed most appropriate for our intervention can be best described by the Behaviour Change Wheel [22] using the following: sources of behavior of psychological capability, reflective motivation, and automatic motivation; intervention functions of education, persuasion, incentivization, and enablement; and policies driven by communication, marketing, and service provision.

### Voice Message Implementation

We are contracting with experts at Innovative Support to Emergencies, Diseases and Disasters (InSTEDD) Southeast Asia to develop and implement the mHealth app for sending targeted phone messages to MoPoTsyo patients. The app “Verboice” is being used to access the information available in the MoPoTsyo database to tailor messages according to the specific characteristics of patients’ disease. Verboice is a free and open-source tool developed by InSTEDD that runs apps through voice, allowing users to listen and record messages in their own language. At the time of informed consent, patients provided 2 current cell phone numbers, their own plus a backup belonging to a family member. Following the development of specific content for message themes identified in the focus groups, electronic capture of data provided by the PEs will allow us to match the specific issues and needs related to hypertension and diabetes management and send relevant messages to individual patients.

### Outcomes

Process outcomes will follow guidelines of the RE-AIM Model [23] to include Reach (how representative is the sample to the targeted population?), Effectiveness (clinical and intermediate outcomes), Adoption (uptake, utilization, and initial implementation), Implementation (fidelity including adherence and delivery), Maintenance (sustainability), as well as acceptability (satisfaction with the intervention). Key clinical outcomes will include control of blood pressure and glucose, medication adherence, use of medical services (laboratory and physician office visits), and improvement in lifestyle factors such as smoking, body mass index, diet, and exercise. Comparisons will be made by the study intervention group at the end of the trial and by changes in clinical outcomes pre- and postintervention period. Furthermore, comparisons will be made for message effectiveness to determine whether certain message topics led to better clinical outcomes.

### Data Management

Data collected by PEs during the pilot study will be entered into the tablet at their homes and will be transferred electronically to the newly enhanced Patient Information System database at MoPoTsyo headquarters. The collected data will be stored on a relational database management system and will be securely backed up to an offsite data center affiliated with MoPoTsyo. All data, however, will become a part of the patients’ medical record for longitudinal tracking and access to clinical staff. These data will be available for inclusion in individual, as well as combined, data reports that are a part of the proposed information system.

All data will be monitored by a trained information techinology staff funded under the program dedicated to overseeing data. He will check for missingness and consistency using preprogrammed algorithms to identify problems. After cleaning, data to be analyzed for outcomes will be deidentified and formatted into analytic files for use by coinvestigators and analysts for evaluation.

### Data Analysis

#### Qualitative Analysis

Audiorecordings from the focus group discussions will be transcribed and translated into English. A subset of transcripts will be back-translated to assure accuracy. The transcripts will be analyzed thematically according to the grounded theory approach. Analysis and interviewing will proceed concurrently so that participant responses and emerging themes can shape future focus group discussions and interviews. A coding scheme will be developed on the basis of the topic guide and emerging themes from the transcripts. Two researchers will independently code all transcripts and compare results. Any discrepancies in coding will be discussed and resolved by the research team. Results will be used to inform researchers of the content of messages within the mHealth app and improve the protocol for the introduction of eHealth communications within this project.

#### Statistical Analyses

Statistical analysis will be performed collaboratively between Cambodian collaborators and the University of Washington to provide experience to them in this activity. All outcomes, both process and clinical, will be described as N (%) or mean (SD) for categorical and continuous data, respectively. Changes in primary study outcomes, including blood pressure, fasting glucose, and medication adherence, will be calculated as the difference from the baseline to 12-month follow-up. We will evaluate study results 3 ways—first as an intention-to-treat comparison of study outcomes associated with patients in the intervention group versus the control group compared by a change in PE overall clinical outcomes for their patients (mean blood pressure, fasting glucose, and medication adherence). In addition, we will conduct an efficiency analysis using linear regression for the study outcomes evaluating group assignment, as well as other variables, calculated at the PE level (ie, % women, mean education, etc). Finally, we will analyze data at the patient level using Generalize Estimating Equations for each outcome evaluating group assignment and clustering data on PE. Primary dependent variables will include changes in the 3 pre and post continuous clinical outcomes—systolic and diastolic blood pressure, fasting blood glucose, and medication adherence as an intermediate outcome. Covariates will include demographics, baseline clinical values, and urban or rural residency of patients. In these analyses, we will use complier-average causal effects (CACE) to look at the efficacy and effectiveness of the mHealth intervention, which takes the clustering effect of PEs, as well as response of patients into consideration. In these patient-level models, we will conduct an intention-to-treat analysis to evaluate the total causal effect of assigning a PE to the mHealth intervention, regardless of compliance. CACE will also allow us to estimate the mHealth intervention’s effectiveness on patient compliers, considering phone messages received and listened to. In CACE, actual treatment received (compliance to the mHealth treatment) becomes a postrandomized variable and effectiveness based on process outcomes (reach and compliance) are addressed. Analyses will be performed using STATA (StataCorp College Station). No interim analyses are planned.

#### Power

We will conduct the primary analysis evaluating mean differences of change in study outcomes by PE group assignment. With a sample of 50 (PE pairwise comparisons between the 2 intervention groups and controls) assuming an alpha of.05 and improvements of controls at a rate similar to 12-month change previously seen, we have 94% power to assess a 50% greater improvement in systolic blood pressure in the intervention group, and 79% power to assess a 40% greater improvement. We will have >99% power to detect differences in efficacy analyses. We believe that these are realistic goals based on results of other rigorous mHealth studies in LMIC [24]. Power calculations were completed using STATA (StataCorp College Station).

## Results

Results of the qualitative research were used to develop the telephone messages according to 6 themes—medications adherence, laboratory testing, physician visits (either annual or for alert determined at PE visit), obesity and weight gain, smoking, and general lifestyle issues (diet, exercise, salt intake, etc). Based on the identified facilitators and barriers, the messages are designed to provide education, motivation, and reminders to help support self-management and address obstacles. Specific scripts were tested with patients and recorded in Khmer by a professional recording studio (Women’s Media Center of Cambodia) using music and voice intonations suggested by advisors.

An algorithm was developed to identify patients needing specific content messages based on the most recent data that were sent electronically to the MoPoTsyo database by the PEs. The delivery of messages was decided according to focus group suggestions, including the use of voicemails rather than text, sending messages at dinnertime or shortly afterward so that a person is at home and can share with family members, limiting frequency to 2-3 messages per week, and eliminating interactive requirements owing to limited access to smartphones and lower cell phone literacy in many rural communities. Results of Verboice tracking allows us to determine which messages successfully reached patients (for later efficacy analyses) and allows the system to send messages additional times when not initially completed.

The focus groups and informant interviews used to develop the mHealth messages were completed during the Spring of 2017. Results of this qualitative work were used to develop the mHealth messages, which were then tested over the summer. Following several revisions of content wording, music, and messenger voice, messages were finalized in July 2017 after which InSTEDD provided channels for delivery of specific themes of message to patients identified by PE data. While the implementation was originally scheduled for 6 months from September 2017 to March 2018, the period was extended 4 additional months because of budgetary efficiencies. Data are currently being cleaned and analyzed. We project that we will have results by Spring 2019.

We plan to disseminate results of this study through workshops and presentations to our Advisory Committee, the Cambodian Ministry of Health (Department of Preventive Medicine), and other health partners (eg, National Institute of Public Health in Phnom Penh) and technology experts (private and public). Furthermore, results will be available in report and presentation format on the website of MoPoTsyo Patient Information Center and the University of Washington Department of Family Medicine. We will use the results of the study to provide evidence-based recommendations to the Ministry of Health as they prepare pilot and other programs and policy for addressing NCDs across Cambodia.

## Discussion

This protocol provides information on a new intervention to develop and test mHealth and eHealth interventions to utilize an existing peer support network for improving hypertension and diabetes management in Cambodia. Our formative work, to develop and pilot-test the messages and distribute the tablets, suggests that his approach may be feasible in other LMIC.

The value of peer support in chronic disease management has been well documented and is a viable alternative when health care infrastructure is inadequate to meet needs [25]. While mHealth messaging programs have rapidly increased in LMIC, evaluations of these programs remain limited and provide inconsistent results [26]. The variety of different features and measured outcomes included in such studies also makes comparisons difficult. While the majority of studies in the literature utilize SMS text messaging, we chose voice messages in our intervention owing to the low literacy rate of our target population. In addition, other mHealth studies in low-resource settings have documented a preference for voice messages by targeted populations. For example, 99% of mothers participating in the Mobile Midwife program in Ghana preferred voice messages over text [27]. Similarly, a study of 488 mobile phone users in India found that 89% preferred to receive medication reminders by voice calls over SMS text messages [28]. Several other studies selecting a voice approach have reported positive results [29-31].

Interactive systems, allowing the recipient to respond to a message on their phone pad, have suggested benefits, although we chose to exclude such a feature as our focus groups informed us that this was confusing to patients. In terms of outcomes, many studies have reported benefits in proxy measures, such as medication adherence, clinic attendance, or behavioral change [30,32-36]. Although improvements in clinical outcomes may be of greatest value in disease control, studies reporting these outcomes are unfortunately less common [13,29,37,38]. Finally, while it is established that mHealth messaging systems involving personalized content are generally more successful [39], personalized interventions are also less common in LMIC, and accessing patient data that are both appropriate and timely may not be possible in many of these settings. As mHealth solutions for electronic health recording and data capture increase, tools for monitoring and accessing timely patient data can provide community health workers, PEs, and providers the information they need to offer relevant and personalized care to patients. Such an approach may be especially useful for managing patients with chronic conditions. This protocol was designed to address these barriers.

While this protocol has many strengths, including the stable infrastructure and PE training already in place by MoPoTsyo, there are a number of limitations, which primarily involve the ability of the targeted messages to reach participants. Problems may involve cell phone network coverage and reliability, cell phone number changes, use of phones across family members, and lack of interest in listening to the messages. Other problems may include the reluctance of PEs to use the tablets in a timely manner, loss of tablets, and factors outside of our control (ie, lack of money to cover medications, etc). While strategies have been developed to minimize these issues, they may still impact the effect of the intervention.

The protocol described here represents an effort to integrate improved data technology for collecting data (eHealth) with individualized voice messaging (mHealth) to encourage self-management of hypertension and diabetes in a low-resource setting. We hypothesize that targeting specific health issues of relevance to patients will be more effective in encouraging good behavior than generic messaging often used in mHealth studies. We hope to show that the promotion of greater communication across providers and patients is feasible in LMIC and will result in better clinical outcomes for their patients.

## Abbreviations

CACE: complier-average causal effects
eHealth: electronic health
InSTEDD: Innovative Support to Emergencies, Diseases and Disasters
LMIC: low- and middle-income countries
mHealth: mobile health
NCD: noncommunicable disease
OD: Operational District
PE: peer educator
SMS: short message service

## Appendix

Multimedia Appendix 1 Peer-reviewer report from the National Institutes of Health.
