# Supplementary material for: Using Targeted mHealth Messages to Address Hypertension and Diabetes Self-Management in Cambodia: Protocol for a Clustered Randomized Controlled Trial
Source: JMIR Res Protoc. 2019 Mar 19;8(3):e11614. doi: 10.2196/11614 (PMC6447150; doi:10.2196/11614)
Supplement: Multimedia Appendix 1 [file resprot_v8i3e11614_app1.pdf]

SUMMARY STATEMENT
(Privileged Communication)

PROGRAM CONTACT:
Laura Povlich
301-496-1653
povlichlk@mail.nih.gov

Release Date: **06/23/2015**

Application Number: **1 R21 TW010160-01**

Principal Investigator

FITZPATRICK, ANNETTE L PHD

Applicant Organization: UNIVERSITY OF WASHINGTON

Review Group: **ZRG1 IMST-K (50)**
Center for Scientific Review Special Emphasis Panel
Mobile Health: Technology and Outcomes in Low and Middle Income Countries

Meeting Date: **06/11/2015**
Council: **AUG 2015**
Requested Start: **09/01/2015**
RFA/PA: **PAR14-028**
PCC: **MHEALTH**
Dual IC(s): **CA, EB, HD, MH**

Project Title: **Integrating Mobile E-Health into Hypertension and Diabetes Management in Cambodia**
SRG Action: **Impact Score: 25**
Next Steps: **Visit http://grants.nih.gov/grants/next_steps.htm**
Human Subjects: **30-Human subjects involved - Certified, no SRG concerns**
Animal Subjects: **10-No live vertebrate animals involved for competing appl.**
Gender: **1A-Both genders, scientifically acceptable**
Minority: **5A-Only foreign subjects, scientifically acceptable**
Children: **3A-No children included, scientifically acceptable**
Clinical Research - not NIH-defined Phase III Trial

Project Year	Direct Costs Requested	Estimated Total Cost
1	125,000	191,054
2	125,000	191,054
TOTAL	250,000	382,107

ADMINISTRATIVE BUDGET NOTE: The budget shown is the requested budget and has not been adjusted to reflect any recommendations made by reviewers. If an award is planned, the costs will be calculated by Institute grants management staff based on the recommendations outlined below in the COMMITTEE BUDGET RECOMMENDATIONS section.

1R21TW010160-01 FITZPATRICK, ANNETTE

RESUME AND SUMMARY OF DISCUSSION: This Exploratory/Developmental Research application from the University of Washington and the MoPoTsyo Patient Information Centre Phnom Penh seeks to implement an enhanced set of features of an existing mHealth communications application for the self-management of hypertension and diabetes, and to pilot test the new system with end-users for acceptability and ease of use. Based on the results of this activity, a randomized controlled clinical intervention study will be run to assess the effectiveness of the application in reducing negative outcomes in diabetes and hypertension patients. The primary reviewers noted that if successful, the ability to reduce mortality from hypertension and diabetes would potentially have a significant impact on public health costs and disease management in Cambodia. Reviewers expressed a moderately high level of enthusiasm for this application with agreement that an outstanding investigative team was seeking to address an important emerging topic in healthcare within Cambodia. Additionally, it was noted that the efforts were well integrated into existing capacity such as pharmacy records and community education programs. However reviewers also expressed concern that there was a lack of Cambodian investigators, that the capacity building within the country was weak, that there too few details describing the randomized comparison, and that the project appeared to be overly ambitious. At the conclusion of the discussion however, enthusiasm for the need to address this emerging health problem, the well-qualified investigative team, and the use of a chronic care model that has been successful in several settings was only somewhat dampened by the weaknesses cited for the design of the randomized trial and the lack of research development within Cambodia. As a consequence, the final potential overall impact of the application was noted to be outstanding.

DESCRIPTION (provided by applicant): The population of Cambodia is rapidly aging. Life expectancy at birth has increased from 58 years in 1995 to 71 years in 2012 driving the rate of previously unknown non-communicable diseases (NCDs) skyward. Ischemic heart disease and stroke are now the top two causes of mortality in this low income country and rates of cardiovascular risk factors including hypertension and diabetes are dramatically increasing. While the Cambodian government has done little to address the growing burden of NCDs afflicting its people, a non-governmental organization (NGO), MoPoTsyo Patient Information Center, has stepped in. Using a model of community-based patient-led Peer Educator Networks, MoPoTsyo is currently delivering self-management training and medications to over 21,000 hypertensive and diabetic patients in Cambodia. In spite of the considerable success this model has demonstrated, control of these conditions – particularly hypertension among non-diabetics - remains a challenge. The primary aim of this application, in response to PAR-14-028, Mobile Health: Technology and Outcomes in Low and Middle Income Countries (R21), is to enhance the communication network between the MoPoTsyo patient database, Peer Educators, pharmacies, and patients, using mobile eHealth tools to activate better compliance with treatment guidelines. Specifically, we plan to 1) Finalize enhanced features of the mHealth communications application for self-management of hypertension and diabetes, and pilot test it with end-users for acceptability and ease of use; 2) Implement a randomized controlled clinical intervention to assess the mHealth communications application in rural and urban-poverty communities for reducing study outcomes related to diabetes and hypertension; and 3) Share results with the Cambodian Ministry of Health and development partners in order to inform health policy makers of study findings and to develop strategies for improving control of hypertension and diabetes throughout Cambodia. This e-health communication system using mobile phone technology will include (a) voice messaging in Khmer allowing illiterate users to participate; (b) interactive tailored reminders and messages to patients for improving support for treatment adherence and goal achievement; (c) integration of messages received and responses from users into the health care database to trigger new messages; (d) access to pharmacy data to monitor individual adherence to medication; (e) access of health care information to Peer Educators to improve monitoring of patients over time; and (f) use of the system to integrate data into the MoPoTsyo database for reports and surveillance of patients. Fifty

Peer Educators representing over 6000 rural, sub-urban, and urban patients will be randomized to the intervention or control. Process outcomes and health indicators including control of blood pressure and glucose, medication adherence, and lifestyle factors such as smoking, body- mass index, diet and exercise, will be evaluated. Results will be used to help develop national health policies addressing the great need for NCD control in Cambodia and serve as a model of other countries in SE Asia.

PUBLIC HEALTH RELEVANCE: This project addresses the critical need for improving hypertension and diabetes control in low income countries where dramatic increases in age and changing economics are driving the onset of these cardiovascular risk factors. We propose to develop and test an eHealth communication system designed to provide interactive voice messages tailored to patients based on recent medical history and treatment adherence to motivate improved self-management. We will evaluate the mobile eHealth system using the Peer Educator Network developed and managed by the non-governmental organization MoPoTsyo in Takeo province and a slum of Phnom Penh to compare approximately 3000 patients receiving the intervention to 3000 receiving usual counseling and care.

CRITIQUE 1:

Significance: 3

Investigator(s): 2

Innovation: 3

Approach: 3

Environment: 2

Overall Impact: This application addresses the important problem of the management of hypertension and diabetes in the Kmer region of Cambodia. The reduction in mortality due to end of the Cambodian civil war and other causes of violent death in the region has increased life-span and a consequent rise in mortality and morbidity from chronic, non-communicative diseases. Progress in improving two important causes (hypertension and diabetes) of this mortality will substantially improve health outcomes in this region of the world, and likely be an important part of economic development. As life expectancy has increased from approximately 58 to 71 years between 1995 and 2012, the prevalence of hypertension has increased to approximately 11% of and will likely continue to increase because of the high-prevalence of pre-hypertension and current under diagnosis.

Diabetes and hypertension are increasingly serious problems in the developing world and in Cambodia in particular. The successful use of WHO guidelines and treatment algorithms in the developed world have substantially reduced the mortality and morbidity of both diseases. Adequate early detection of diabetes allows the use of effective and inexpensive medications for early stage disease (e.g., metformin), delaying the need for more expensive insulin support. Peer educators have the potential for immediate and sustained improvement in patient understanding of the disease and of the value of treatment. Because of the infrastructure present in the MoPoTsyo Patient Information Centre and the relatively straightforward technology being used, this project is likely to have large impact.

1. Significance:

Strengths

- This application addresses the important problem of the management of hypertension and diabetes in the Kmer region of Cambodia.

- The project accounts for the varying literacy of the population, by using voice messaging to remind participants of medication and upcoming visits
- The system seems well integrated into the best of the existing capacity in Cambodia, including the project link with pharmacy records and a set of Peer educators.
- The system to be implemented is modeled on a chronic care model (CCM) that has been successful in several settings and has been studied by WHO.
- The project will partner with MoPoTsyo Patient Information Center, a Cambodian non-governmental organization (NGO) for people with chronic disease in Cambodia.
- The roll-out consists of a well-conceived set of steps to calibrate the system with both the needs of the participants and cultural context. Aim 1 will use focus groups of patients (or potential patients) to help gauge usability of the messaging and Peer educator systems.
- Aim 1 also includes pre-testing of the technology by peer educators, hopefully avoiding the problem of dropping developed country technology into a very different environment.
- Aim 2 includes an important randomized study comparing outcomes of the mHealth system with individuals receiving only peer counseling.
- Aim 3 cites intent to share results with the Cambodian Ministry of Health to attempt to maximize the impact of the study on health policy in the region.
- Generally, the investigators seem to have made a careful study of the reasons similar initiatives have not worked in the past, and have outlined steps to reduce the chance of failure, including 1) use of an established Peer Educator Network; 2) accessing patient data to tailor electronic messages to patients; 3) adding an interactive component for tracking and recording responses; 4) capability to send messages to contacts of patient non-responders; 5) the use of using tablet-based; 6) maintaining sustainability of the hypertension/diabetes management program by increasing patient volume at pharmacies. (summarized from the grant)

Weaknesses

- Perhaps the most substantial weakness is the lack of detail about the randomized comparison. Without clear evidence of either the superiority of the mHealth initiative or its lack of a differential effect, the decision of whether to expand the initiative in a resource poor environment will be a difficult one. The trial will use a cluster randomized design, with 25 peer educators in the mHealth group and 25 using the usual approach. It is unclear from the proposal whether the primary analysis will be based on mean outcomes for each peer, or on patient level analysis. If the analysis is based on a patient level analysis (as the section on data analysis implies), there are many important details missing from the power calculation, including assumptions about the intra-cluster correlation for patients with the same peer counselor. The description in the proposal does not discuss the possibility of cross cluster contamination when patients who may live or work near each other are randomized to different groups. These missing details make it difficult for this reviewer to evaluate the potential success of the trial. This reviewer is aware of and sensitive to the constraints of the length allowed for R21 proposals, but with some judicious editing of redundant discussions more detail could have been squeezed in to the proposal.
- If successful, the randomized trial will provide information about the differential effect of the mHealth initiative compared to usual peer educator approach, but it will not provide information about the generalizability of the results unless the participant population is reasonably representative of the population at large. I did not find much information about how the participants will be chosen, except that they will be limited to the Takeo region.

2. Investigator(s):

Strengths

- This is a strong team that is well-qualified to conduct the research. The PI (A. Fitzpatrick) is a research professor at the University of Washington, with appointments in family medicine, epidemiology and global health. She a particular research interest in cardiovascular disease and has extensive experience LIMCS, having led previous projects in Nepal.
- The co-investigators (Thompson and LoGerfo) are eminently well-qualified to participate in the research.
- The collaborating investigators from Cambodia all seem well-qualified, although except for van Pelt, their role seems limited to consultant with funding to cover only 7 days a year for each.

Weaknesses

- The important role of data quality control seems to be in the hands of a student assistant. With the right student, that may well work out, but in my experience, this very important aspect of a large and complex study should be carried out by a professional with the skills for both electronic checking of data records and the experience to recognize when components of a data collection system might not be functioning properly.
- The collaborating statistical group is well qualified for the work, but it is not clear how involved they were in the preparation of the proposal.

3. Innovation:

Strengths

- This is an innovative project in several respects: (1) it integrates several approaches to insuring success, including learning from both patients and peer counselors how this technology might be adapted in Cambodia; (2) it builds on the strength of the NGO MoPoTsyo in patient level interaction; (3) it uses current and widely available mobile phone technology; and (4) includes the potential of evaluation through a randomized comparision. While the particular technology used in the project may not be leading edge, its integration with other aspects of the project is impressive.

Weaknesses

- None Noted

4. Approach:

Strengths

- The approach to the research is well-thought out. The use of focus groups to measure perceptions and usability of self-help methods and key informant interviews with the peer educators will provide key information in the overall design.
- The randomized trial estimating the effectiveness of the mHealth initiative is a strong component of the approach, though some details on sample size and power need clarification.

Weaknesses

- None Noted

5. Environment:

Strengths

- The University of Washington is well-suited to support this work, and the MoPoTsyo Patient Information Centre is ideally suited for an in-country site. UW has a strong program in preventive health, and the MoPoTsyo Center has a well-established cohort of peer educators.

Weaknesses

- None Noted

Research Capacity Building:

Strengths

- If successful, the project will increase the in-country capacity for patient self-management of disease and for data collection about hypertension and diabetes in Cambodia. Long term, this may well lead to increased understanding of the prevalence of pre-diabetes and hypertension, allowing policy questions to be examined in a more evidence-based setting.

Weaknesses

- The computing and other equipment will remain in Cambodia after the project ends, and the peer educators will be better equipped to use this equipment, it is less clear from the application that staff in country will participate in the intellectual components of the research.

Protections for Human Subjects: Acceptable Risks and/or Adequate Protections

- Adequate, except for monitoring plan. See details in the review

Data and Safety Monitoring Plan (Applicable for Clinical Trials Only):

Unacceptable

- o The project will include a randomized trial of mHealth vs usual peer counseling. Strictly speaking, this is not a randomized treatment trial, but monitoring in this setting still seems prudent, if only to insure interpretability of data and to be sure that the design goals are being met.

Inclusion of Women, Minorities and Children:

- Sex/Gender: Distribution justified scientifically
- Race/Ethnicity: Distribution justified scientifically
- Inclusion/Exclusion of Children under 21: Excluding ages < 21 justified scientifically

Vertebrate Animals: Not Applicable (No Vertebrate Animals)

Biohazards: Not Applicable (No Biohazards)

Resource Sharing Plans: Acceptable

Budget and Period of Support: Recommend as Requested

CRITIQUE 2:

Significance: 3

Investigator(s): 6

Innovation: 4

Approach: 3

Environment: 4

Overall Impact: This purpose of this study is to enhance the communication network between the patient database maintained by an NGO (MoPoTsyo) , Peer Educators, pharmacies, and patients, using mobile eHealth tools to activate better compliance with treatment guidelines. The investigators plan to 1) Finalize enhanced features of the mHealth communications application for self-management of hypertension and diabetes, and pilot test it with end-users for acceptability and ease of use; and 2) Implement a randomized controlled clinical intervention to assess the mHealth communications application in rural and urban communities for reducing study outcomes related to diabetes and hypertension. Diabetes and hypertension is a growing public health problem in Cambodia and developing a mHealth tool to combat the burden associated with these diseases is important.

This proposal has the potential to be incorporated within the national policy of Cambodia, if found to be effective, and will contribute to the control of diabetes and hypertension. Methods described to develop and evaluate the mHealth application are well described and feasible.

1. Significance:

Strengths

- The study is significant as this will utilize the existing healthcare delivery system, provided by an NGO, to facilitate service delivery for hypertension and diabetes.
- The existence of peer educators (PEs) and the use of PEs to reinforce the use of mHealth tools to manage chronic diseases could play a key role in the self-management of hypertension and diabetes.
- The investigators reviewed and demonstrated the effectiveness of mobile phone based intervention to address chronic disease epidemic and highlighted the need for an mHealth based program in Cambodia. If successful, such program will reduce the system related and personal barriers to promote the control of hypertension and diabetes.

Weaknesses

- None Noted

2. Investigator(s):

Strengths

- The PI has assembled a team of researchers with expertise in clinical NCD care, IT engineering and system design, epidemiology, health economics, and community involvement to assure timely completion of the project.
- The PI is experienced in the field and had conducted several NIH-funded studies including one in SE Asia (i.e. in Vietnam).

Weaknesses

- Only one foreign site investigator is included who himself is non-local, highlighting the lack of engagement of developing country partner in the study.

3. Innovation:

Strengths

- The use of patient data on health metrics and medication adherence to tailor electronic messages to individual patients; and adding an interactive voice component for response to messages that can be tracked and incorporated into the parent database, is innovative.

Weaknesses

- mHealth tool have been used in many developing countries to manage chronic diseases, but it may be new for the Cambodian health systems.

4. Approach:

Strengths

- The proposed approach, to collaborate with MoPoTsyo Patient Information Center in Cambodia to enhance their Peer Educator Network model addressing hypertension and diabetes management through application of mHealth tailored phone messaging and improved eHealth communication throughout the system, is appropriate.
- The use of interactive voice messaging system is useful to reach those illiterate users and would be useful for many rural settings.
- Inclusion of an advisory committee, including members from multiple stakeholders, is useful to monitor the progress of the study.

Weaknesses

- The gender imbalance in the reach of subjects through PEs is not discussed. Of the total 6070 subjects, only 2000 are male. Is there a reason for such discrepancies?
- The accomplishment of the aims as stated within the 24 month period may be a too tight.

5. Environment:

Strengths

- University of Washington has adequate facilities to carry out the proposed study.

Weaknesses

- However, it is not very clear whether the institutional environment of MoPoTsyo Patient Information Centre Phnom Penh will be supportive to carry out the proposed work.

Research Capacity Building:

Strengths

- There is potential to build capacity of the foreign investigators, however, given the fact that only one investigator is named in the application the impact may be minimal.

Weaknesses

- It is not clear how the collaboration between the HIC and LMIC will be sustained.

Protections for Human Subjects: Acceptable Risks and/or Adequate Protections

Data and Safety Monitoring Plan (Applicable for Clinical Trials Only):

Not Applicable (No Clinical Trials)

Inclusion of Women, Minorities and Children:

- Sex/Gender: Distribution justified scientifically
- Race/Ethnicity: Distribution justified scientifically
- Inclusion/Exclusion of Children under 21: Excluding ages < 21 justified scientifically

Vertebrate Animals: Not Applicable (No Vertebrate Animals)

Biohazards: Not Applicable (No Biohazards)

Resource Sharing Plans: Not Applicable (No Relevant Resources)

Budget and Period of Support: Recommend as Requested

CRITIQUE 3:

Significance: 2

Investigator(s): 1

Innovation: 2

Approach: 2

Environment: 1

Overall Impact: Heart disease and stroke are leading causes of mortality in Cambodia. MoPoTyso (NGO) has ongoing program for delivering self-management and training for hypertensive and diabetic patients in Cambodia. This proposal seeks to provide an mHealth tool to enhance and customize this self-management and training program to patients for improved efficacy and to capture key data for surveillance and reporting. Objective health outcomes blood pressure and glucose control, medication adherence, and lifestyle factors will be compared across a a randomized study of 3000 patients receiving standard counseling versus the mHealth initiative. Data and results are shared with the Ministry of Health to adapt national policy. If effective, this model system may be applied to other disease care and across the region.

1. Significance:

Strengths

- Addresses major public health problem in Cambodia

- Electronic data capture will increase ability to monitoring patients, health outcomes, and creating reports of outcomes and impact.
- Incorporate peer educators to increase acceptability of program design and implementation
- Tests if tailored messaging to patients if improve medication adherence

Weaknesses

- None

2. Investigator(s):

Strengths

- Senior research faculty in collaboration with extensive field research experience, funding, and publications.
- Collaboration has long history with prior joint research and publications
- Clear administrative structure and support for study team at MoPoTyso.
- Consulting faculty with National Institute for Public Health support research activities

Weaknesses

- None

3. Innovation:

Strengths

- New technical ability will permit electronic data capture for monitoring patients, health outcomes, and creating reports of outcomes and impact.
- New technology permits tailored messaging to patients to improve medication adherence

Weaknesses

- None

4. Approach:

Strengths

- Formation of advisory committee beneficial to this complex and large collaboration
- Clear data management and analysis plan
- Focus group design and analysis appropriate
- Randomized trial powered to detect at least a 40% change in blood pressure
- Multiple outcomes of interest can be evaluated.

Weaknesses

- Scientific justification for minimum 40% change in primary outcome not addressed.

5. Environment:

Strengths

- Excellent facilities listed at both University of Washington and MoPoTyso collaboration.

Weaknesses

- None

Research Capacity Building:

Strengths

- Equipment and software technology will transfer to MoPoTyso local NGO
- Program planned to continue after grant period
- Designed as model program for region
- Builds upon a strong existing collaboration

Weaknesses

- None

Protections for Human Subjects: Acceptable Risks and/or Adequate Protections

Data and Safety Monitoring Plan (Applicable for Clinical Trials Only):

Not Applicable (No Clinical Trials)

Inclusion of Women, Minorities and Children:

- Sex/Gender: Distribution justified scientifically
- Race/Ethnicity: Distribution justified scientifically
- Inclusion/Exclusion of Children under 21: Excluding ages < 21 justified scientifically
- disease topic not applicable to children

Vertebrate Animals: Not Applicable (No Vertebrate Animals)

Biohazards: Not Applicable (No Biohazards)

Resource Sharing Plans: Acceptable

Budget and Period of Support: Recommend as Requested

CRITIQUE 4:

Significance: 3

Investigator(s): 3

Innovation: 2

Approach: 2

Environment: 3

Overall Impact: This project is significant because it addresses the growing burden of chronic disease in LMICs. It has the potential to impact national health policies toward NCD management in Cambodia. The co-morbidity of hypertension and diabetes is common and this approach acknowledges more than one 'disease' to be addressed, but 'diseases' that share many components of NCD management. The proposal builds on a successful program of diabetes and hypertension peer education conducted by MoPoTsyo center. The effectiveness of mobile phones to improve health outcomes will be measured qualitatively as well as conducting a randomized controlled trial to test the acceptability of using mobile phones. The innovation of this project is the multiple dimensions of an ehealth communications system (a-f in the abstract). The proposal presents a very detailed explanation of the mobile technology and approaches the application of ehealth as an integrated and comprehensive health information system for managing chronic conditions. The project has gathered well-equipped team of experts with the PI having extensive research experience relevant to the project goals, and the CO-I from Cambodia has a long history of collaborative research and clinical innovations in diabetes care.

1. Significance:

Strengths

- The project addresses the growing burden of chronic conditions in LMICs.
- Peer education model will be evaluated to assess effectiveness for providing a broader scope of supportive care for people with the common comorbidity of diabetes and hypertension, and will have a significant impact in a region with a serious shortage of healthcare providers.

Weaknesses

- None Noted

2. Investigator(s):

Strengths

- The PI has extensive experience in LMIC working on cardiovascular health and mobile ehealth communications.
- Co-investigator from Cambodia has a long and successful record of providing peer support for diabetes management and conducting collaborative research with international partners.

Weaknesses

- None Noted

3. Innovation:

Strengths

- The strategy of using peers for NCD management addresses the reality of a severe shortage of qualified healthcare workers and evidence is growing on the effectiveness of this model of chronic care management.
- The Aim 3 plan to specifically share results directly with policy makers and the ministry of health improves the likelihood that effective strategies will be sustainable.

Weaknesses

- None Noted

4. Approach:

Strengths

- Phase 1 will consist of qualitative data obtained from patients and peer educators and Phase 2 will be an RCT to compare the effectiveness of mhealth phone messaging and regular peer education counseling.

Weaknesses

- Outcomes are more intermediate outcomes of BP and diabetes control as well as health behaviors, and not health outcomes of reduced morbidity or mortality, although these would require longer term intervention to assess

5. Environment:

Strengths

- MoPo Tsyo center has a long history of success in improving control of persons with diabetes through a peer educator program

Weaknesses

- None Noted

Research Capacity Building:

Strengths

- None Noted

Weaknesses

- None Noted

Protections for Human Subjects: Acceptable Risks and/or Adequate Protections

- Human subject protections assured

Data and Safety Monitoring Plan (Applicable for Clinical Trials Only):

Acceptable

- data security and sharing plans are clearly articulated

Inclusion of Women, Minorities and Children:

- Sex/Gender: Distribution justified scientifically
- Race/Ethnicity: Distribution justified scientifically
- Inclusion/Exclusion of Children under 21: Excluding ages < 21 justified scientifically
- I did not see a specific inclusion criterion for age, other than the statement that 60 adults will be recruited and that the prevalence of diabetes and hypertension increases with age.

Vertebrate Animals: Not Applicable (No Vertebrate Animals)

Biohazards: Not Applicable (No Biohazards)

Resource Sharing Plans: Acceptable

- Described in the proposal

Budget and Period of Support: Recommend as Requested

THE FOLLOWING SECTIONS WERE PREPARED BY THE SCIENTIFIC REVIEW OFFICER TO SUMMARIZE THE OUTCOME OF DISCUSSIONS OF THE REVIEW COMMITTEE, OR REVIEWERS' WRITTEN CRITIQUES, ON THE FOLLOWING ISSUES:

PROTECTION OF HUMAN SUBJECTS (Resume): ACCEPTABLE

The four issues concerning protections of human subjects were adequately addressed and risks were noted to be minimal. Samples and data to be used will be de-identified and there were no concerns.

INCLUSION OF WOMEN PLAN (Resume): ACCEPTABLE

The application stated that both genders will be adequately recruited; this was justified and therefore noted to be scientifically acceptable.

INCLUSION OF MINORITIES PLAN (Resume): ACCEPTABLE

The application stated that only foreign subjects will be recruited; this was justified and therefore noted to be scientifically acceptable.

INCLUSION OF CHILDREN PLAN (Resume): ACCEPTABLE

The application stated that children will not be recruited for this study; this was justified and therefore noted to be scientifically acceptable.

COMMITTEE BUDGET RECOMMENDATIONS: The budget was recommended as requested.

NIH has modified its policy regarding the receipt of resubmissions (amended applications). See Guide Notice NOT-OD-14-074 at http://grants.nih.gov/grants/guide/notice-files/NOT-OD-14-074.html. The impact/priority score is calculated after discussion of an application by averaging the overall scores (1-9) given by all voting reviewers on the committee and multiplying by 10. The criterion scores are submitted prior to the meeting by the individual reviewers assigned to an application, and are not discussed specifically at the review meeting or calculated into the overall impact score. Some applications also receive a percentile

ranking. For details on the review process, see
http://grants.nih.gov/grants/peer_review_process.htm#scoring.

MEETING ROSTER

Center for Scientific Review Special Emphasis Panel
CENTER FOR SCIENTIFIC REVIEW
Mobile Health: Technology and Outcomes in Low and Middle Income Countries
ZRG1 IMST-K (50) R
June 11, 2015 - June 12, 2015

CHAIRPERSON

LIECHTY, EDWARD A, MD
PROFESSOR
DEPARTMENT OF PEDIATRICS
INDIANA UNIVERSITY
SCHOOL OF MEDICINE
INDIANAPOLIS, IN 462025210

SPRUIJT-METZ, DONNA D, PHD
RESEARCH PROFESSOR AND DIRECTOR
USC MHEALTH COLLABORATORY
CENTER FOR ECONOMIC AND SOCIAL RESEARCH
UNIVERSITY OF SOUTHERN CALIFORNIA
LOS ANGELES, CA 90089

MEMBERS

ABDULLAH, ABU SALEH, MD, PHD
ASSOCIATE PROFESSOR
DEPARTMENT OF GENERAL INTERNAL MEDICINE
BOSTON MEDICAL CENTER
BOSTON UNIVERSITY MEDICAL CAMPUS
BOSTON, MA 02118

ABROMS, LORIEN C ABROM, SCD
ASSOCIATE PROFESSOR
MILKEN INSTITUTE SCHOOL OF PUBLIC HEALTH
DEPARTMENT OF PREVENTION AND COMMUNITY HEALTH
GEORGE WASHINGTON UNIVERSITY
WASHINGTON, DC 20037

AHAMED, SHEIKH I, PHD
PROFESSOR
DEPARTMENT OF MATHEMATICS, STATISTICS
AND COMPUTER SCIENCE
MARQUETTE UNIVERSITY
MILWAUKEE, WI 53233

ASCHE, CARL , PHD
DIRECTOR AND PROFESSOR
CENTER FOR OUTCOMES RESEARCH
UNIVERSITY OF ILLINOIS COLLEGE OF MEDICINE
PEORIA, IL 61605

BARCLAY, GILLIAN R, DDS
PUBLIC HEALTH ADVISORS
GLOBAL PARTNERSHIPS FOR DEVELOPMENT
PONTIAC, MI 48340

BAUERMEISTER, JOSE A, MPH, PHD
ASSOCIATE PROFESSOR
HEALTH BEHAVIOR AND HEALTH EDUATION
UNIVERSITY OF MICHIGAN SCHOOL OF PUBLIC HEALTH
ANN ARBOR, MI 48109

BAUMANN, LINDA J, PHD
PROFESSOR
UNIVERSITY OF WICONSIN
SCHOOL OF NURSING
MADISON, WI 53792

BENNETT, IAN MOORE, MD, PHD
ASSOCIATE PROFESSOR
DEPARTMENT OF FAMILY MEDICINE
AND COMMUNITY HEALTH
UNIVERSITY OF PENNSYLVANIA HEALTH SYSTEM
PHILADELPHIA, PA 19104

BOIVIN, MICHAEL J, MPH, PHD
PROFESSOR
DEPARTMENTS OF PSYCHIATRY AND
NEUROLOGY/OPHTHALMOLOGY
COLLEGE OF OSTEOPATHIC MEDICINE
MICHIGAN STATE UNIVERSITY
EAST LANSING, MI 48824

CUPERTINO, PAULA
ASSOCIATE PROFESSOR
DEPARTMENT OF PREVENTIVE MEDICINE
AND PUBLIC HEALTH
UNIVERSITY OF KANSAS MEDICAL CENTER
KANSAS CITY, KS 66160

DE ERAUSQUIN, GABRIEL A, MD, PHD
PROFESSOR AND DIRECTOR
PSYCHIATRY, NEUROLOGY AND NEUROSURGERY
ROSKAMP CHAIR OF BIOLOGICAL PSYCHIATRY
MORSANI COLLEGE OF MEDICINE
UNIVERSITY OF SOUTH FLORIDA
TAMPA, FL 33613

DECKELBAUM, RICHARD J, MD
PROFESSOR AND DIRECTOR
DEPARTMENT OF PEDIATRICS
INSTITUTE OF HUMAN NUTRITION
COLUMBIA UNIVERSITY
NEW YORK, NY 10032

DEPP, COLIN A, PHD
ASSOCIATE PROFESSOR
DEPARTMENT OF PSYCHIATRY
SCHOOL OF MEDICINE
UNIV. OF CALIFORNIA, SAN DIEGO
LA JOLLA, CA 92093

EHIRI, JOHN E, MPH, PHD
PROFESSOR AND DIRECTOR
DIVISION OF HEALTH PROMOTION SCIENCES
MEL AND ENID ZUCKERMAN COLLEGE OF PUBLIC HEALTH
UNIVERSITY OF ARIZONA
TUCSON, AZ 85724

EHRHARDT, STEPHAN , MPH, MD
ASSOCIATE PROFESSOR
DEPARTMENT OF EPIDEMIOLOGY
JOHNS HOPKINS SCHOOL OF PUBLIC HEALTH
BALTIMORE, MD 21205

ESSIEN, EKERE J, MPH, MD, DRPH
PROFESSOR
DEPARTMENT OF CLINICAL SCIENCES
AND ADMINISTRATION
COLLEGE OF PHARMACY
UNIVERSITY OF HOUSTON
HOUSTON, TX 77030

GANCE-CLEVELAND, BONNIE , PHD
LORETTA FORD PROFESSOR
DIVISION OF WOMEN, CHILDREN, AND FAMILY HEALTH
COLLEGE OF NURSING
ANSCHUTZ MEDICAL CAMPUS
UNIVERSITY OF COLORADO
AURORA, CO 80045

GIORDANI, BRUNO , PHD
PROFESSOR
DEPARTMENT OF PSYCHIATRY, NEUROLOGY, &
PSYCHOLOGY
SCHOOL OF NURSING
UNIVERSITY OF MICHIGAN
ANN ARBOR, MI 48109

HARRINGTON, DAVID P, PHD
PROFESSOR
DEPARTMENT OF BIOSTATISTICS AND STATISTICS
HARVARD UNIVERSITY & DANA-FARBER CANCER
INSTITUTE
BOSTON, MA 02115

HEFFRON, RENEE ANNETTE, MPH, PHD
DEPARTMENT OF GLOBAL HEALTH
THE UNIVERSITY OF WASHINGTON
SEATTLE, WA 98104

INTILLE, STEPHEN S, PHD
ASSOCIATE PROFESSOR
COLLEGE OF COMPUTER AND INFORMATION SCIENCE
AND BOUVE COLLEGE OF HEALTH SCIENCES
NORTHEASTERN UNIVERSITY
BOSTON, MA 02115

LELUTIU-WEINBERGER, CORINA , PHD
RESEARCH SCIENTIST
DEPARTMENT OF PSYCHOLOGY
HUNTER COLLEGE OF THE CITY
UNIVERSITY OF NEW YORK
NEW YORK, NY 10065

LEMMA, WULETA
ASSOCIATE PROFESSOR
DEPARTMENT OF GLOBAL COMMUNITY HEALTH
AND BEHAVIORAL SCIENCES
SCHOOL OF PUBLIC HEALTH AND TROPICAL MEDICINE
TULANE UNIVERSITY
NEW ORLEANS, LA 70112

LIU, LONGJIAN , MD, PHD
ASSOCIATE PROFESSOR
DEPARTMENT OF EPIDEMIOLOGY AND BIOSTATISTICS
DREXEL UNIVERSITY
PHILADELPHIA, PA 19102

LUQUE, JOHN S, MPH, PHD
ASSOCIATE PROFESSOR
DEPT. OF COMMUNITY HEALTH BEHAVIOR & EDUCATION
JIANN-PING HSU COLLEGE OF PUBLIC HEALTH
GEORGIA SOUTHERN UNIVERSITY
STATESBORO, GA 30458

MARIENFELD, CARLA B, MD
ASSISTANT PROFESSOR
DEPARTMENT OF PSYCHIATRY
YALE UNIVERSITY SCHOOL OF MEDICINE
NEW HAVEN, CT 06511

MURPHY, ROBERT L., MD
PROFESSOR AND DIRECTOR
CENTER FOR GLOBAL HEALTH
DEPARTMENT OF MEDICINE
FEINBERG SCHOOL OF MEDICINE
NORTHWESTERN UNIVERSITY
CHICAGO, IL 60611

MURRAY, MEGAN B, MPH, SCD, MD
PROFESSOR
DEPARTMENT OF EPIDEMIOLOGY
SCHOOL OF PUBLIC HEALTH
HARVARD UNIVERSITY
BOSTON, MA 02115

NACHEGA, JEAN B, MPH, MD, PHD
ASSOCIATE PROFESSOR
DEPARTMENT OF EPIDEMIOLOGY
SCHOOL OF PUBLIC HEALTH
UNIVERSITY OF PITTSBURGH
PITTSBURGH, PA 15261

POELLABAUER, CHRISTIAN , PHD
ASSOCIATE PROFESSOR
UNIVERSITY OF NOTRE DAME
NOTRE DAME, IN 46556

RAM, PAVANI K, MD
ASSOCIATE PROFESSOR
SCHOOL OF PUBLIC HEALTH
EPIDEMIOLOGY AND ENVIRONMENTAL HEALTH
DIRECTOR, OFFICE OF GLOBAL HEALTH INITIATIVES
STATE UNIVERSITY OF NEW YORK BUFFALO
BUFFALO, NY 14214

STCLAIRE, TAMARA
CHIEF INNOVATION OFFICER
XEROX COMMERCIAL HEALTHCARE
SACRAMENTO, CA 95837

STEINHUBL, STEVEN R, MD
DIRECTOR
DIGITAL MEDICINE
SCRIPPS TRANSLATIONAL SCIENCE INSTITUTE
LA JOLLA, CA 92037

THOMAS, JAMES C, MPH, PHD
ASSOCIATE PROFESSOR
DEPARTMENT OF EPIDEMIOLOGY
UNIVERSITY OF NORTH CAROLINA AT CHAPEL HILL
CHAPEL HILL, NC 275997400

TISCH, DANIEL J, MPH, PHD
ASSOCIATE PROFESSOR
SCHOOL OF MEDICINE
CASE WESTERN RESERVE UNIVERSITY
CLEVELAND, OH 44106

TORIOLA, ADETUNJI T, MPH, MD, PHD
ASSISTANT PROFESSOR
DEPARTMENT OF SURGERY
DIVISION OF PUBLIC HEALTH SERVICES
WASHINGTON UNIVERSITY SCHOOL OF MEDICINE
ST. LOUIS, MO 63110

WADE, ERIC , PHD
ASSISTANT PROFESSOR
DEPARTMENT OF MECHANICAL, AEROSPACE,
AND BIOMEDICAL ENGINEERING
UNIVERSITY OF TENNESSEE
KNOXVILLE, TN 37966

WALANI, SALIMAH R, MPH, MSN, PHD
DIRECTOR
GLOBAL HEALTH PROGRAMS
MARCH OF DIMES
WHITE PLAINS, NY 10605

WALSH, JULIA A, MD
ADJUNCT PROFESSOR
PUBLIC HEALTH
MATERNAL & CHILD HEALTH
UNIVERSITY OF CALIFORNIA BERKELEY
BERKELEY, CA 94720

XIAN, XIAOJUN , PHD
ASSOCIATE RESEARCH SCIENTIST
CENTER FOR BIOELECTRONICS AND BIOSENSORS
THE BIODESIGN INSTITUTE
ARIZONA STATE UNIVERSITY
TEMPE, AZ 85287

YOUNGBLOOD, G. MICHAEL
SENIOR AI SYSTEMS ARCHITECT AND RESEARCHER
INTERACTIVE INTELLIGENCE AREA
INTERACTION AND ANALYTICS LAB
PALO ALTO RESEARCH CENTER
PALO ALTO, CA 94304

MAIL REVIEWER(S)

AMAYA-BURNS, ALBA P, MD
ASSOCIATE PROFESSOR
DUKE KUNSHAN UNIVERSITY
JIANGSU, 215316
CHINA

FINKEL, MADELON , PHD
PROFESSOR
DEPARTMENT OF PUBLIC HEALTH
WEILL CORNELL MEDICAL COLLEGE
NEW YORK, NY 10065

KIENE, SUSAN MARIA, MPH, PHD
ASSOCIATE PROFESSOR
DIVISION OF EPIDEMIOLOGY AND BIOSTATISTICS
DEPARTMENT OF GLOBAL HEALTH
GRADUATE SCHOOL OF PUBLIC HEALTH
SAN DIEGO STATE UNIVERSITY
SAN DIEGO, CA 92182

KIRBY, BRIAN J, PHD
ASSOCIATE PROFESSOR
SIBLEY SCHOOL OF MECHANICAL & AEROSPACE ENG
COLLEGE OF ENGINEERING
KIRBY RESEARCH GROUP
CORNELL UNIVERSITY
ITHACA, NY 14853

MURUGESAN, MALLESH
PRESIDENT AND CEO
ABEYON
MIAMI, FL 33156

STELLING, JOHN , MPH, MD
CO-DIRECTOR
COLLABORATING CENTRE FOR SURVEILLANCE
OF ANTIMICROBIAL RESISTANCE
WORLD HEALTH ORGANIZATION
BOSTON, MA 02115

SCIENTIFIC REVIEW OFFICER

RICHON, ALLEN , PHD
SCIENTIFIC REVIEW OFFICER
CENTER FOR SCIENTIFIC REVIEW
NATIONAL INSTITUTES OF HEALTH
BETHESDA, MD 20892

EXTRAMURAL SUPPORT ASSISTANT

AKOMAH, STEPHEN
LEAD EXTRAMURAL SUPPORT ASSISTANT
CENTER FOR SCIENTIFIC REVIEW
NATIONAL INSTITUTES OF HEALTH
BETHESDA, MD 20892

Consultants are required to absent themselves from the room during the review of any application if their presence would constitute or appear to constitute a conflict of interest.
